# Supplementary material for: TOCOSH FLOUR (Solanum tuberosum L.): A Toxicological Assessment of Traditional Peruvian Fermented Potatoes
Source: Foods. 2020 Jun 2;9(6):719. doi: 10.3390/foods9060719 (PMC7353511; doi:10.3390/foods9060719)
Supplement: Supplementary file 1 [file foods-09-00719-s001.pdf]

Supplementary material

TOCOSH FLOUR (*Solanum tuberosum* L.): A toxicological Assessment of traditional Peruvian fermented potatoes

Jonas Roberto Velasco-Chong [1], Oscar Herrera-Calderón [1,*], Juan Pedro Rojas-Armas [2], Renán Dilton Hañari-Quispe [3], Linder Figueroa-Salvador [4], Gilmar Peña-Rojas [5], Vidalina Andía-Ayme [6], Ricardo Ángel Yuli-Posadas [7], Andres F. Yepes-Perez [8] and Cristian Aguilar [9]

1 Academic Department of Pharmacology, Bromatology and Toxicology, Faculty of Pharmacy and Biochemistry, Universidad Nacional Mayor de San Marcos, Jr Puno 1002, Lima 15001, Peru; jvelasco9490@gmail.com

2 Department of Dynamic Sciences, Faculty of Medicine, Universidad Nacional Mayor de San Marcos, Av. Miguel Grau 755, Cercado de Lima 15001, Peru; jprojasarmas@yahoo.com

3 Clinical Pathology Laboratory, Faculty of Veterinary Medicine and Zootechnics, Universidad Nacional del Altiplano, Av Floral 1153, Puno 21001, Peru; rhanari@unap.edu.pe

4 School of Medicine, Faculty of Health Sciences, Universidad Peruana de Ciencias Aplicadas, Prolongación Primavera 2390, Lima 15023, Peru; pcmelfig@upc.edu.pe

5 Laboratory of Cellular and Molecular Biology, Biological Sciences Faculty, Universidad Nacional de San Cristóbal de Huamanga, Portal Independencia 57, Ayacucho 05003, Peru; gilmar.pena@unsch.edu.pe

6 Food Microbiology Laboratory, Biological Sciences Faculty, Universidad Nacional de San Cristóbal de Huamanga, Portal Independencia 57, Ayacucho 05003, Peru; vidalina.andia@unsch.edu.pe

7 Universidad Continental, Av San Carlos 1980, Huancayo 12000, Peru; ryuli@continental.edu.pe

8 Chemistry of Colombian Plants, Institute of Chemistry, Faculty of Exact and Natural Sciences, University of Antioquia-UdeA, Calle 70 52–21, A.A 1226, Medellin 050010, Colombia; andresf.yepes@udea.edu.co

9 Laboratory of Pathology, Instituto Nacional Cardiovascular, Jirón Coronel Zegarra 417, Jesús María 15072, Peru, a.crisaguilar@gmail.com

* Correspondence: oherreraca@unmsm.edu.pe; Tel.: +51-956-550-510

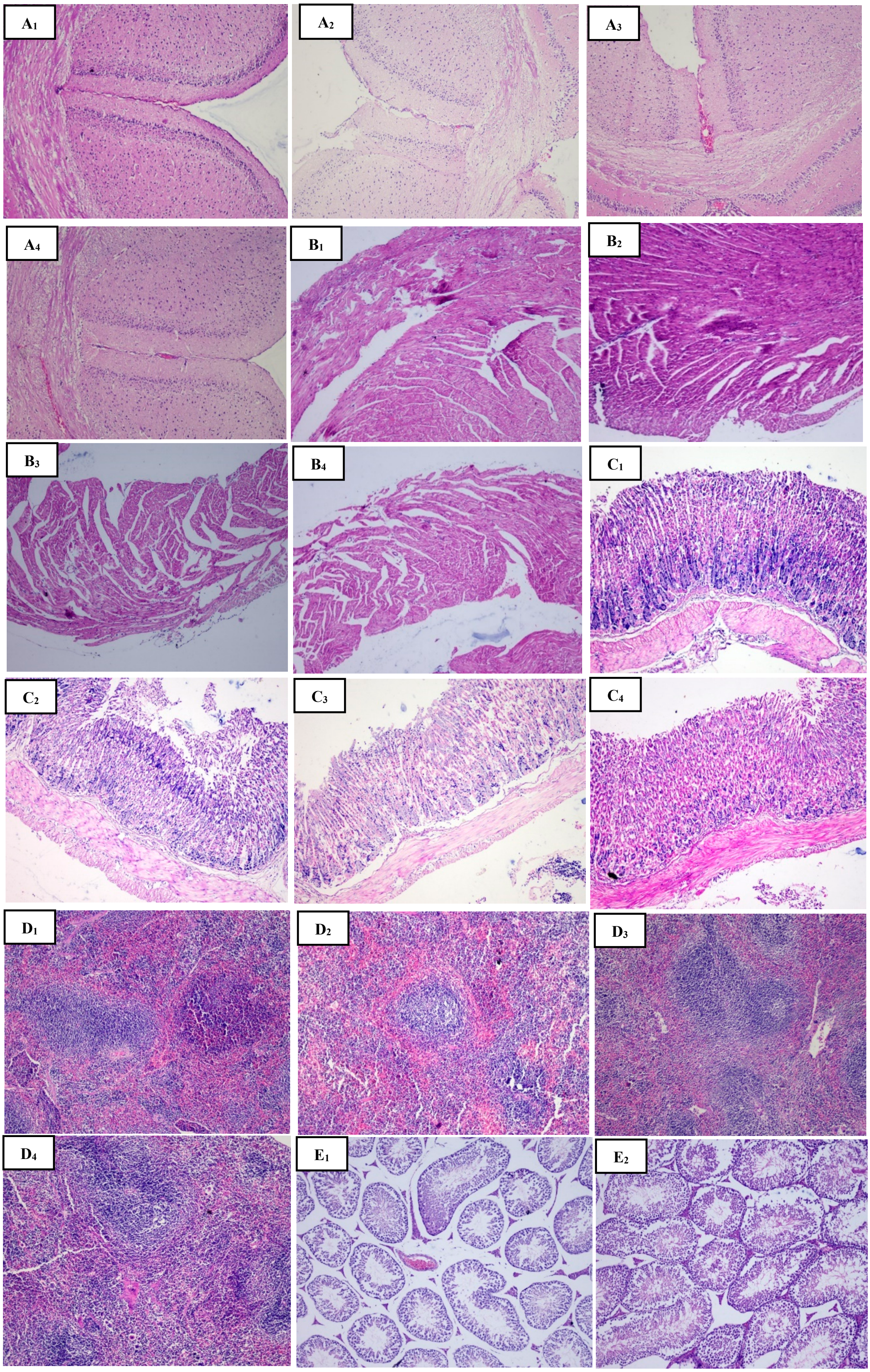
A1
A2
A3
A4
B1
B2
B3
B4
C1
C2
C3
C4
D1
D2
D3
D4
E1
E2

Figure S1. Images of some tissues of male and female mice without specific alterations that received fixed doses of 2000 mg/kg and 5000 mg/kg of Tocosh Flour stained with H&E and 40X.
A1. Male mouse at the dose of 2000 mg / kg. Brain - Brain parenchyma without significant alterations.
A2. Female mouse at the dose of 2000 mg / kg. Brain - Brain parenchyma without significant alterations.
A3. Male mouse at the dose of 5000 mg / Kg. Brain - Brain parenchyma without significant alterations.
A4. Female mouse at the dose of 5000 mg / Kg. Brain - Brain parenchyma without significant alterations.
B1 Male mouse at the dose of 2000 mg / Kg. Heart - Myocardium without specific alterations.
B2 Female mouse at the dose of 2000 mg / Kg. Heart - Myocardium without specific alterations.
B3 Male mouse at the dose of 5000 mg / Kg. Heart - Myocardium without specific alterations.
B4 Female mouse at the dose of 5000 mg / Kg. Heart - Myocardium without specific alterations.
C1. Male mouse at the dose of 2000 mg / Kg. stomach - Gastric mucosa without affected alterations.
C2 Female mouse at the dose of 2000 mg / Kg. stomach - Gastric mucosa without affected alterations.
C3 Male mouse at the dose of 5000 mg / Kg. stomach- Gastric mucosa without affected alterations.
C4. Female mouse at the dose of 5000 mg / Kg. stomach - Gastric mucosa without affected alterations.
D1 Male mouse at the dose of 2000 mg / kg. Spleen - splenic parenchyma without specific alterations.
D2 Female mouse at the dose of 2000 mg / Kg. Spleen - splenic parenchyma without specific alterations.
D3 Male mouse at the dose of 5000 mg / Kg. Spleen - Splenic parenchyma without specific alterations.
D4 Female mouse at the dose of 5000 mg / Kg. Spleen - splenic parenchyma without specific alterations.
E1 Male mouse at the dose of 2000 mg / Kg. Testis - Testicular parenchyma without specific alterations.
E2 Male mouse at the dose of 5000 mg / Kg. Testis - Testicular parenchyma without specific alterations

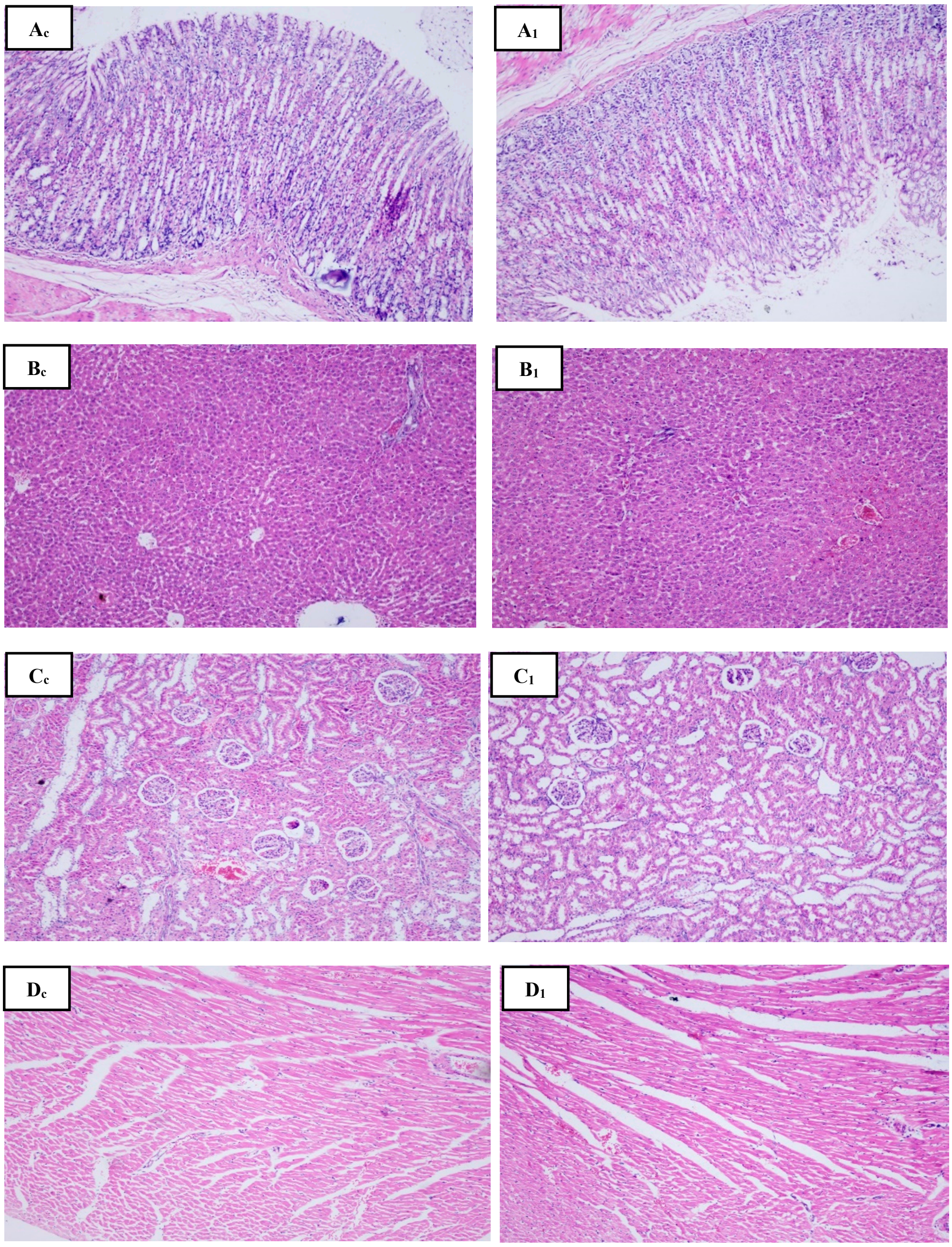

Figure S2. Images of the organ sections: Stomach (A), liver (B), Kidney (C) and heart (D), of male rats who were administered Tocosh flour for 28 days. Subscript C and 1 refer to the control group and the group administered at dose of 1000 mg / kg /day, respectively. H&E staining, 40X.

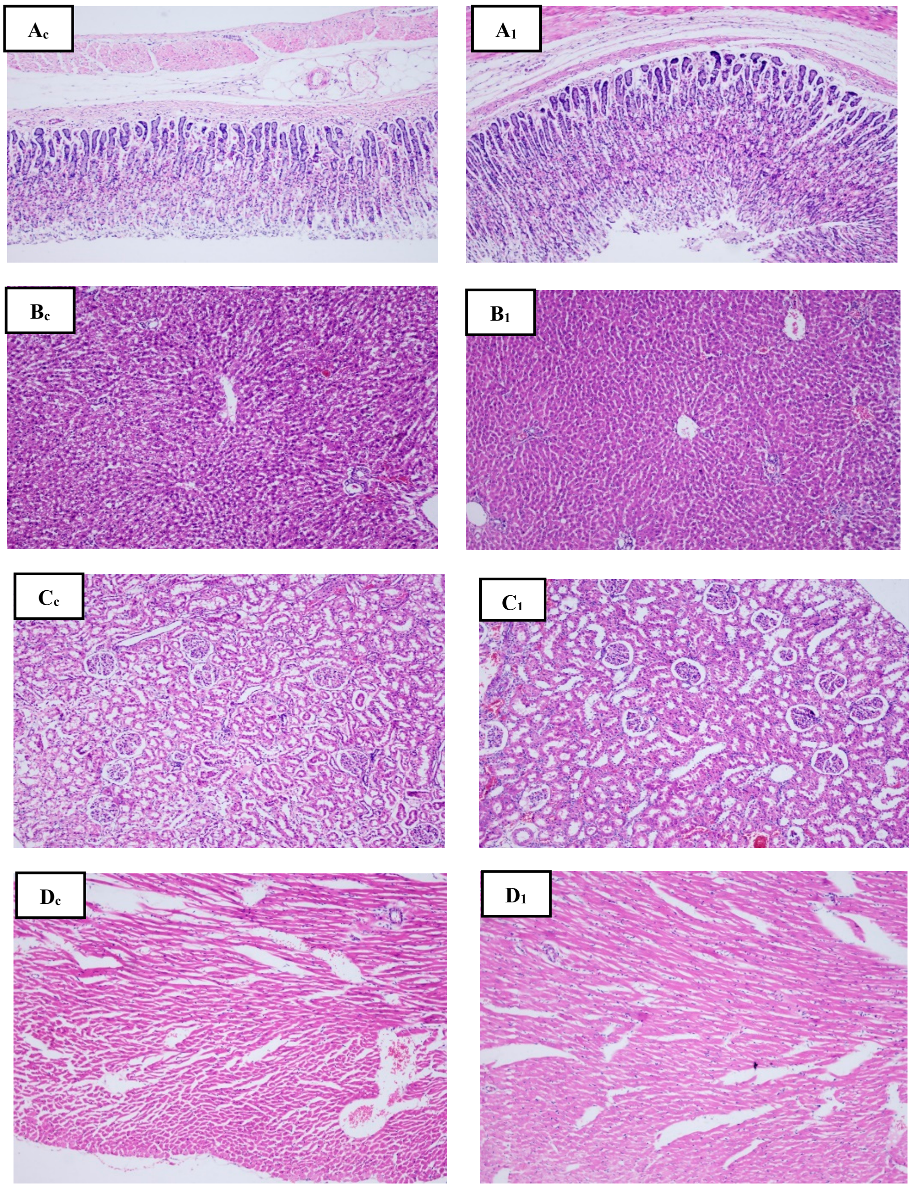

Figure S3. Images of the organ sections such as: Stomach (A), liver (B), Kidney (C) and heart (D), of female rats that were administered Tocosh flour for 28 days. Subscript C and 1 refer to the control and group administered at dose of 1000 mg / kg / day, respectively. H&E staining, 40X.
